# What is the impact of the UK soft drinks industry levy on childhood tooth decay?

**DOI:** 10.1038/s41432-024-01025-3

**Published:** 2024-06-06

**Authors:** Samantha Watt

**Affiliations:** School of Clinical Dentistry, University of Sheffield, Sheffield, S10 2TA UK

**Keywords:** Nutrition and diet in dentistry, Paediatric dentistry

## Abstract

**Design:**

Interrupted time series analysis.

**Data analysis:**

An interrupted time series (ITS) analysis was conducted to determine if there was an association between the announcement and implementation of the soft drinks industry levy (SDIL) and rates of hospital admission for tooth extractions due to dental caries in children. Hospital Episode Statistics (HES) were used on hospital admissions for tooth extraction of one or more primary or permanent tooth due to a primary diagnosis of dental caries in children aged 0–18 years attending a National Health Service (NHS) hospital in England from January 2012 (pre-SDIL) to February 2020 (post-SDIL implementation). HES data were grouped and summarised by Index of Multiple Deprivation (IMD) and age group.

**Results:**

There was an absolute reduction of 3.7% (95% CI 5.3% to 2.2%) per 100,000 population/month and a relative reduction of 12.1% (95% CI 17.0% to 7.2%) in hospital admissions for carious tooth extractions in all children (0–18 years) compared if there had been no announcement of the SDIL (counterfactual scenario). Reductions were observed in children living in most areas regardless of the level of deprivation and most notably in the youngest children (<10 years).

**Conclusions:**

An ITS analysis of administrative data on hospital admissions found the announcement of the UK SDIL was associated with improvements (reduction) in the incidence of hospital admissions for tooth extractions due to dental caries. This study provides evidence of benefits of the UK SDIL to children’s oral health.

## A Commentary on

**Rogers N T, Conway D I, Mytton O** **et al**.

Estimated impact of the UK soft drinks industry levy on childhood hospital admissions for carious tooth extractions: interrupted time series analysis. *BMJ Nutr Prev Health* 2023; 10.1136/bmjnph-2023-000714.

**GRADE Rating:** 

## Commentary

Sugar consumption is a key aetiological factor for dental caries. A range of approaches are required to reduce consumption of sugar and improve oral health from chairside prevention delivered by dental teams to upstream public health policy such as taxation of sugar^1,2^. This paper has focused on the soft drinks industry levy (SDIL), an upstream fiscal measure, the study was well-designed and a welcome addition to the evidence-base regarding the impact of fiscal measures on the health of children and young people, however, this study was observational and under GRADE’s scoring system is classed as low quality.

This study highlights the positive impact the SDIL has had on the oral health of children, particularly young children. In addition, adds to the evidence-base regarding the impact of the UK SDIL on population health. The SDIL was announced in March 2016, it aimed to reduce the prevalence of childhood obesity, and was implemented in April 2018^3^. It was two-tiered levy which was designed to incentivise manufacturers to reformulate high sugar soft drinks to move them to a lower tier. Manufacturers were subject to a charge £0.24/ litre of soft drinks containing more than 8 g sugar per 100 ml, £0.18 for 5–8 g sugar per 100 ml and no levy for less than 5 g.

Tooth extractions due to dental caries are the most common reason for hospital admission in children aged 5 to 9 years^4^. Hospital admissions for decay-related extractions in children aged 0 to 19 years cost the NHS £40.7 million in the financial year 2022 to 2023^4^. An ITS analysis was conducted to determine if the SDIL resulted in a change in hospital admissions for extractions due to dental caries in children from January 2012 (pre-SDIL) to February 2020 (post-implementation of the SDIL). Over the 98-month study period in children aged 0–18 years, there was a relative reduction in hospital admissions for carious tooth extractions. Of interest Rogers and colleagues found that the greatest impact on admissions and oral health occurred between the SDIL announcement (March 2016) and implementation (April 2018) due reformulation in advance of implementation of the levy.

Reductions in hospital admissions were observed in children living in most areas, regardless of the level of deprivation (all quintiles except the middle (IMD3) quintile). Modelling studies had predicted that sugar sweetened beverage taxes would lead to the greatest reduction in caries in the lowest income groups^5^. However, the authors suggested that they did not observe a greater reduction in the most deprived groups (IMD 1 and 2) as water fluoridation is more common in deprived areas of England^6^. Of interest, a more recent modelling study published by the same team predicted that the SDIL will reduce inequalities in dental caries in children and young people but this may only be seen longer term^7^.

The authors used HES data for the number of extractions in children undertaken in hospitals due to dental caries, they acknowledge that not all hospital providers of this service appear on the HES dataset. However, the numbers of providers remained consistent during the study period. An exploration of the locations of the hospital providers not appearing on the HES dataset and the demography of the populations they serve is warranted to determine if this could have impacted the findings of this study.

Reductions in admissions were observed for children aged 0–4 years and 5–9 years, however no significant changes in children in the 10–14 and 15–18-years age groups were found. The authors hypothesise that this was due to this group having more autonomy over their diet. Therefore, there is a need for future interventions developed specifically for 10–18 years olds to reduce their need for caries-related admissions and dental teams are well placed to deliver such interventions. However, while behaviour change interventions have been developed to promote other oral health behaviours, it is noted in the evidence-based toolkit for prevention ‘Delivering Better Oral Health’ that there is very little quality evidence about effective interventions delivered by dental teams to reduce sugar consumption^8^. Further research is required to develop evidence-based behaviour change interventions for sugar reduction to complement further upstream measures to improve the oral health of (older) children.

Overall, the success of the SDIL in reducing the number of hospital admissions for extraction of teeth due to dental caries in children is an encouraging step to improving children’s oral and general health. The benefits of the SDIL on children’s health should strengthen the case to support other public health measures such as restrictions on food marketing, advertising and promotions targeted at young people, further expanding the SDIL to include other sugar sweetened beverages such as sugary milk drinks and restricting the sale of caffeinated energy drinks. The limited impact of the SDIL on hospital admissions for older children also emphasises the need for more evidence-based behaviour change interventions delivered by dental teams to help reduce sugar consumption for this specific group.

Practice points

- This study adds to the growing evidence base of the benefits of the SDIL on the health of children and young people.
- Additional public health measures such as expansion of the levy and restrictions on the marketing and sale of high-sugar food and beverages are required to reduce inequalities and improve the (oral) health of children and young people.
- Further research is required to develop evidence-based interventions, to be delivered by dental teams, aimed at reducing dietary sugar consumption in children and young people.
